# Traffic Accidents in Children and Adolescents: A Complex Orthopedic and Medico-Legal Approach

**DOI:** 10.3390/children10091446

**Published:** 2023-08-24

**Authors:** Ștefan Popa, Carmen Iulia Ciongradi, Ioan Sârbu, Ovidiu Bîcă, Irene Paula Popa, Diana Bulgaru-Iliescu

**Affiliations:** 1 2nd Department of Surgery–Pediatric Surgery and Orthopedics, “Grigore T. Popa” University of Medicine and Pharmacy, 700115 Iași, Romania; stefan.popa@umfiasi.ro (Ș.P.); sarbu.ioan@umfiasi.ro (I.S.); ovidiu-daniel.bica@umfiasi.ro (O.B.); 2 Department of Physiology, “Grigore T. Popa” University of Medicine and Pharmacy, 700115 Iași, Romania; 3 3rd Department of Medical Specialities–Legal Medicine, “Grigore T. Popa” University of Medicine and Pharmacy, 700115 Iași, Romania; diana.bulgaru@umfiasi.ro

**Keywords:** road traffic accidents, children, adolescents, orthopedic approach, fractures, medico-legal approach, safety

## Abstract

Traffic accidents involving children and adolescents present complex challenges from both the medico-legal and orthopedic standpoints. Despite the implementation of road traffic safety laws, pediatric road traffic injuries continue to be a significant contributor to mortality rates, physical harm, and hospitalization on a global scale. For children and young people, automobile accidents are considered to be the primary culprit of mortality in developed nations. Even in highly developed nations, trauma is a significant factor in infant mortality. Each age category, from childhood to young adulthood, has its fracture patterns, as their skeletons are considerably different from those of adults. The consequences of traffic accidents extend beyond the immediate physical trauma. The medico-legal aspects surrounding these incidents add another layer of complexity, as legal repercussions may affect the responsible adult or parent, particularly in cases involving child fatalities. To effectively address traffic accidents in children and adolescents, a comprehensive approach is necessary. This approach should involve not only medical professionals but also legal experts and policymakers. Collaboration between orthopedic specialists, medico-legal professionals, law enforcement agencies, and relevant government bodies can facilitate the development and implementation of strategies aimed at prevention, education, the enforcement of traffic laws, and improved infrastructure. By addressing both the medical and legal aspects, it is possible to enhance road safety for children and adolescents, reducing the incidence of injuries and their associated long-term consequences. In this review, we aimed to summarize traffic accidents in children and adolescents from a complex orthopedic and medico-legal approach.

## 1. Introduction

Road traffic accidents (RTAs) represent a significant and escalating contributor to mortality rates, physical harm, and hospitalization on a global scale [1]. The impact of road traffic trauma and fatalities on health and financial expenditures is highly substantial. Annually, the global number of fatalities resulting from RTAs is estimated to be around 1.35 million [2,3]. The 2018 WHO Global Status Report on Road Safety indicates a notable disparity in the prevalence of road traffic fatalities among countries, with low-/middle-income countries (LMIC) constituting 90% of the aforementioned deaths [4]. A considerable number of fatalities on the road are attributed to vulnerable road users, such as pedestrians and cyclists [2]. Pedestrian motor vehicle collisions (PMVCs) pose considerable danger to children and youth pedestrians between the ages of 2 and 20, due to their limited developmental ability to perceive road and traffic hazards [5]. Due to their small stature, children are particularly susceptible to serious injuries and mortality. In the course of 2016, the global number of pedestrian fatalities among individuals aged 0–20 years was estimated to be around 72,000 [2]. Skeletal injuries are seldom potentially fatal, but they can cause serious complications [6,7]. Pediatric orthopedic surgeons are frequently heavily engaged, since 76% of children with multiple injuries experience extremity trauma [8]. In addition, the occurrence of skeletal trauma, such as spine, clavicle/scapula, femur, and pelvic trauma, raises the need for intensive care and the length of hospitalization [9,10]. Therefore, the medico-legal approach to traffic accidents in children and adolescents is an important area of study that aims to understand the legal and medical aspects of such accidents and their impact on young individuals. This comprehensive analysis will discuss various factors related to medico-legal approaches, including the outcomes of traffic accidents in children and adolescents, guidelines for personal injury assessment, risk factors associated with severe injuries, pattern injuries to pedestrians, and factors affecting pedestrian injuries.

In this review, we aimed to summarize traffic accidents in children and adolescents from a complex orthopedic and medico-legal approach.

## 2. Epidemiology

Traffic accidents constitute a significant urban health issue. Injuries caused by traffic collisions are an important contributor to mortality, hospitalization, and impairment among children worldwide [11]. For children and young people, automobile accidents are considered to be the primary culprit of mortality in developed nations [12,13]. The highest proportion of anticipated lasting impairment and the need for emergency surgery were associated with road traffic injuries [14]. The majority of research focuses on epidemiological findings, probable socio-economic hazards, and the pathomechanism of accidental mortality across young individuals [15,16]. Over 1.5 million cases of pediatric trauma take place every year in the United States, leading to roughly 600,000 admissions to hospitals and 15,000–20,000 pediatric fatalities [17]. The proportion of pedestrian victims (64.9%) exceeded that of vehicle occupants (14.9%) [18,19,20]. According to various research studies, a large number of pedestrians who experience grievous injuries in motor vehicle collisions are children [21,22,23]. A worldwide effort to reduce deadly traffic accidents has been implemented since 1979, for every demographic category [24,25]. In accordance with a yearly assessment by the Organization for Economic Co-operation and Development, the total amount of road fatalities decreased by 15% between 2010 and 2014, a decline comparable to that seen between 2006 and 2010. Nonetheless, this downward pattern continues to fluctuate. Furthermore, it was reported in January 2016 that road fatalities increased by 2.4% in 2015, for the second year in a row, whereas the percentage of trauma incidents decreased by 3.5% [25]. In Latin America, road collisions account for 1.43% of all infant and child fatalities and 13.83% of all fatalities among children between the ages of 5 and 14. A prior investigation on infant and childhood road safety in Panama revealed that road-related mortality in infants and children is an important health issue, with the largest percentage of fatal outcomes documented for children under the age of five. Globally, the average yearly motor-vehicle-collision-related fatality rate (per million people) for 0 to 14 year olds is 6, while in Latin America, it is 32 [26].

**Figure 1:**
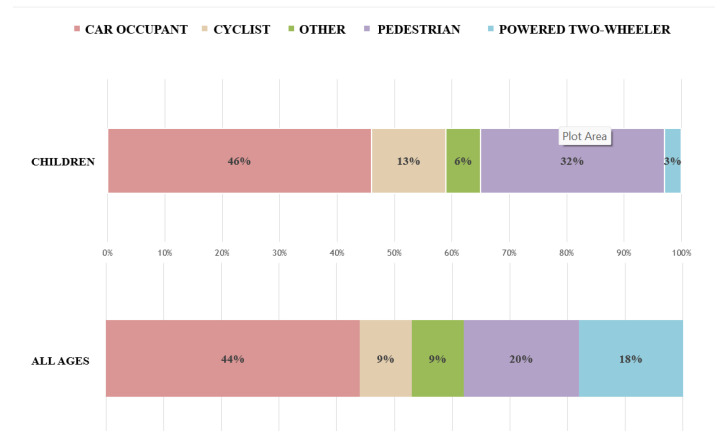
Fatalities among children–transport mode differences (2020). Source: CARE, EUROSTAT.

**Figure 2:**
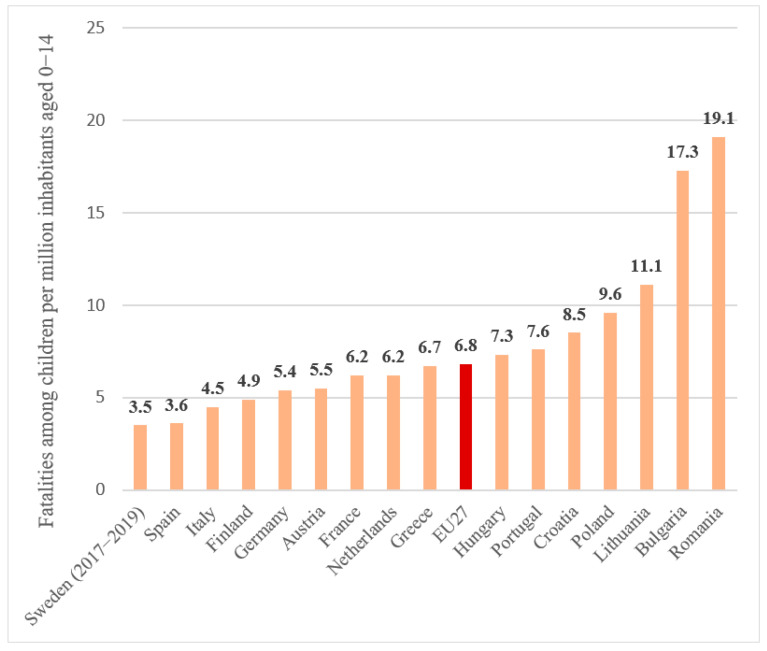
Fatalities among children per million inhabitants aged 0–14 per country in the EU27 (2018–2020). Source: CARE, EUROSTAT.

In accordance with the European Road Safety Observatory [27], the overall total of road casualties among minors dropped by 47% between 2011 and 2020; the most significant reduction in mortality was recorded among those aged 5 to 9 years, because of increased parental supervision, safety education programs, and improved road safety measures. With regards to means of transportation, children experienced a disproportionately elevated mortality rate among those using the most susceptible modes: 32% were pedestrians, while 13% were cyclists (Figure 1). The percentages in question are EU medians, but several EU nations have significantly greater rates. In Romania, roughly one out of every two minor deaths was a pedestrian. In the Netherlands, 47% of juvenile casualties involved cyclists. In accordance with the mortality measurement (deaths per million of the population), the eastern EU countries performed the lowest. The majority of these nations were ranked higher when the percentage of minors fatally wounded in relation to the overall amount of road casualties was used as the measurement. This indicates that the high road fatality rate for minors in these countries is associated with the high road fatality rate for all road users, irrespective of age. However, there were also countries with higher-than-average scores regarding both indicators, including Romania, Bulgaria, and Latvia (Figure 2).

## 3. The Orthopedic Approach to Traffic Accidents in Children and Adolescents

Even in highly developed nations, trauma is a significant factor in infant mortality [27]. Each age category, from childhood to young adulthood, has its fracture patterns, as their skeletons are considerably different from those of adults [28]. Several pediatric injuries to the musculoskeletal system are uncommon, while some of them are often misdiagnosed and mistreated, whereas others, particularly those addressed from peripheral institutions, may be neglected. These types of injuries have been characterized as exceedingly rare, or atypical among minors in the available research. It is uncertain the extent to which this information may reflect a single instance or a widespread but underappreciated occurrence [29].

Fracture patterns in accidents are frequently age or stage-of-growth specific. Consequently, it is essential to comprehend the standard range of injuries that children experience as they grow. Uncertainty surrounds the right evaluation required to foresee prospective atypical injuries. A healthcare professional can determine the probable nature of described injury mechanisms based on the understanding of typical mechanisms for any particular fracture pattern and pertinent medical data. A physician’s evaluation of a child with orthopedic trauma starts with an exhaustive record of the beginning and development of injury-related manifestations, meticulously documented. It ought to incorporate all current and past traumatic occurrences, as well as any health issues [29]. Examining the patient’s overall health along with vital signs; recording any injuries; exposing all impacted areas, all extremities, and joints; and pointing out any deformity, edema, tenderness, and limitation in mobility restrictions should comprise the physical assessment. It is required to have good knowledge of the physiology and anatomy of the human body to effectively treat patients with trauma brought on by RTAs. Rare pediatric traumas and fractures require a thorough clinical and radiological assessment to prevent underdiagnosis, which could be the outcome of a postponed presentation, an incoherent medical record, or numerous wounds, continuing to be related to medical disorders around the world [29]. Orthopedic management should prioritize preserving the limb in cases of severe open fractures, through prompt treatment, splinting limbs throughout initial resuscitation, enabling transfers to radiology rooms and intensive care units, minimizing pain, and promoting early mobilization. Children have been endowed with an immense ability for rehabilitation, and the orthopedic treatment of skeletal trauma ought to be vigorous in order to maximize limb recovery. When dealing with a multiple injury, the use of plaster cast as a noninvasive treatment is determined by the presence or absence of skin wounds. Nevertheless, it is advisable to steer clear of options like hip spica or extended limb casts whenever possible, due to the prolonged immobilization duration and resulting joint stiffness. Generally, elastic stable intramedullary nailing (ESIN) and plating are the gold standard in dealing with diaphyseal fractures nowadays. For comminuted open fractures, that are contaminated, one option could be the external fixators. They are not disruptive with the final therapy when they are indicated correctly while keeping the permanent implant (nail or plate) in mind. They are simple to assemble and can therefore be applied quickly. They lower pain, provide a way through incisions, facilitate recurrent neurovascular examinations, and can be employed as “portable” traction if the patient needs to be moved across the hospital. The incompatibility of ferromagnetic metallic fixators with MRI is an important limitation of their use. Nonferromagnetic, MRI-compatible external fixators are feasible but high-priced. On certain occasions, if the construct is solid and the fracture is properly aligned, a fixator might be used as the final procedure [30].

### 3.1. Cervical Spine Care

The pediatric cervical spine stands apart from the mature spine due to its growing skeleton, insufficient formation of the spinous processes, ligament laxity, the rather straight alignment of the facet joints, a significant head-to-body proportion, and underdeveloped musculature. The resulting variations result in distinct injury patterns, and the outcome tends to be favorable [31].

The concepts of Advanced Trauma Life Support (ATLS^®^) extend to the initial care of a pediatric patient suffering from cervical trauma. At the location of the accident, immobilization in a neutral posture needs to be conducted and sustained as long as the child receives a complete examination in the hospital’s emergency room. The immobilization process avoids the situation that happens when instability is first missed and a neurological deficiency arises throughout the child’s movement as an outcome. Children with potential injuries to the neck were previously positioned on a spinal board and a hard cervical collar was put on, along with sandbags or blocks on both sides of their head and tape to fixate and immobilize the head [32]. Stiff collars, on the other hand, frequently fail to suit children properly, particularly those who are extremely young. Furthermore, young people tend to feel anxious and in distress, rendering collar placement challenging and sometimes hazardous. The most secure technique is pragmatic, enabling the infant to adopt a posture that feels natural and granting manual in-line stability. If an adequate-sized collar can be securely and effectively fitted, this is suitable at this point; on the other hand, just preserving a neutral or relaxed posture with blocks/rolled-up towels placed on both sides of the head and tape to hold them positioned is sufficient. NICE and the advanced pediatric life support program have both recommended employing this method [32,33]. It is additionally essential to be cautious of the possibility that in these situations, a cervical collar could exacerbate atlanto-occipital displacement and enhance brain damage. If the aforementioned pattern of damage becomes apparent, merely sandbags and tape ought to be employed [34]. Because an infant or young child’s head is proportionately considerably bigger in contrast to his/her body than an adult’s, resting supine on a spinal board forces the head toward a minor degree of flexion. To offset this impact, an occipital recess or thoracic rising could be required [35]. In the event of uncertain trauma, unconsciousness, torticollis, cervical musculoskeletal stiffness, important painful sensations in the neck, or neurological signs/symptoms that could be temporary or long-lasting (radiculopathy/myelopathy according to the nature of the trauma), cervical spine harm ought to be taken into account. In the context of trauma, children are far less probable than adults to develop neurological impairment, and partial damage to the spinal cord is more prevalent (75% incomplete vs. 25% complete) [36]. Total spinal cord damage, which results in the impairment of sensory and motor abilities beneath the point of injury, is frequently permanent and carries severe implications.

Given the slight excessive movement of the spine, cervical spine fractures in children younger than two years of age are uncommon, but cervical cord injuries are common. The skeletal spinal column is capable of sustaining two inches of elongation, whilst the neurovascular structures rupture at a quarter-inch strain [37]. This leads to spinal cord lesions with no radiological abnormalities (SCIWORA). Nevertheless, SCIWORA is not frequent, and a magnetic resonance imaging (MRI) examination is required for identifying this condition [38,39]. Between the ages of 2 and 8, trauma develops at or above the C3 level. In this age category, the center of the movement is located at C2–C3, in contrast to adults, where it is at C5–C6. Since cervical spine injuries are common in crashes involving vehicles (both occupant and pedestrian) as well as in falling from a height, an in-depth examination of the cause of wounds ought to be documented. Frequently, children are not able to offer a complete medical record, and indicators of lesions might be modest. In the event of doubt, the cervical spine should undergo extensive assessment. Conventional X-rays covering the cervical spine are among the most widely acknowledged primary imaging techniques. Between 20 and 25 percent of fractures are susceptible to being missed with a cross-table lateral exposure solely; therefore, an anteroposterior image is mandatory to detect adjacent mass and transverse process injuries. The odontoid view is ineffective in children younger than eight years old, and its application continues to be debatable [32,34,35]. Images that fail to depict the whole cervical vertebrae should be rejected. Several typical patterns in the cervical spine of children, including pseudo subluxation at C2–C3 and retropharyngeal enlargement resulting from crying, may deceive the physician; therefore, the guidance of a pediatric radiologist should be considered. The utilization of computed tomography (CT) scans routinely in infants continues to be disputed since it poses the potential hazard of them receiving an excessive amount of radiation to the thyroid gland. However, in cases where a child becomes unconscious or cannot be assessed neurologically within 72 h, the initial investigation that is promptly carried out is an MRI. This is particularly important for unconscious children and situations where understanding potential neurological issues is challenging [40].

### 3.2. Open Fractures

Open fractures are rare and tend to be worsened by injury to soft tissues and compartment syndrome [41]. In 2000, Rennie et al. analyzed the distribution and prevalence of fractures in children younger than 16 years old who presented to healthcare facilities in Edinburgh, Scotland. They discovered 2198 fractures in 2168 patients. The prevalence rate was 20.2 per 1000 children per year. Assessing the place of occurrence of fractures revealed that 82.2% occurred in the upper limb, 17.3% in the lower limb, and 0.5% in the pelvis or spine, with 0.7% of all fractures being open [42]. Court-Brown et al. reported that around 22 percent of young pedestrians that were involved in vehicle accidents experienced fractures, whereas 40% of adult pedestrians experienced fractures in a comparable form of crash. The reason for this is due to the likelihood that children are prone to “bounce” after being struck [43]. Ten percent of high-velocity multi-trauma victims sustain open fractures [6,7]. Throughout the world, open fractures are categorized using the revised Gustilo–Anderson classification [44,45]. Additional classifications have been suggested [46,47], yet the modified Gustilo–Anderson classification continues to be the most prevalent, despite not being child-specific. Nevertheless, the concepts regarding proper emergency debridement, skeletal stabilization, and the handling of soft tissues are similarly relevant to this demographic [47,48,49]. In every instance of open fractures, debridement of the wound should preferably be performed by an orthopedic surgeon working alongside a plastic surgeon [50,51]. The present research suggests that wound debridement is not required for open fractures of Type I in minors [52,53]. Debridement within six hours of trauma is not acknowledged in scientific research nor is it always attainable [54,55]. The latest guidelines state that it ought to be performed in the first 24 h following the trauma of except of extensive contamination, devascularization of the limb, or compartment syndrome (CS), in which circumstance it needs to be performed immediately [30].

As indicated by Table 1, open fractures represent a challenge due to systemic and local complications.

#### 3.2.1. Open Forearm Bone Fractures

Open fractures of the forearm and humerus are uncommon as evidenced by the paucity of the literature, but usually entail an excellent outcome if conventional open fracture treatment principles are followed [56].

#### 3.2.2. Open Femoral Fractures

**Figure 3:**
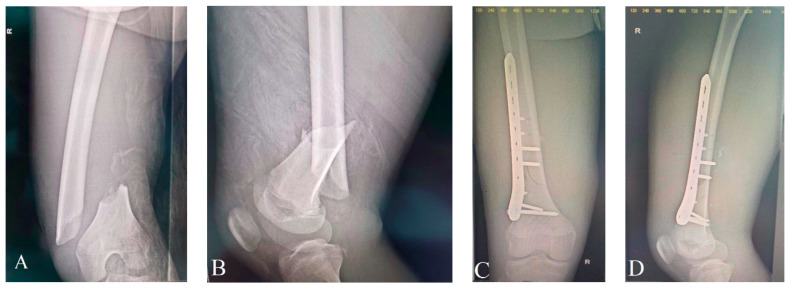
Fifteen-year-old boy involved in a car accident (pedestrian) who suffered a Gustilo–Anderson type I right distal femur fracture, picture (**A**,**B**), which was surgically resolved by open reduction and osteosynthesis with a distal locked femoral plate picture (**C**,**D**) at “St. Mary’s” Clinical Emergency Hospital for Children, Iași, Romania, Pediatric Orthopedics Department.

Open femur fractures are unusual and indicative of high-velocity trauma. They frequently come together with additional injuries, especially head trauma, and the child needs to be evaluated thoroughly to rule out these types of injuries [8,57,58]. In the instance of resuscitation, external fixation of the fractured femur is becoming more frequently utilized as a preventative approach [59]. Debridement of an open femoral fracture is identical to that among all open fractures. In the majority of instances, thigh muscles offer sufficient protection, and surgical treatment is scarcely required. The fracture is definitively fixed immediately when the child is secure. According to the patient’s age, fracture setting, accessible resources, and level of competence, there exists several treatment options, such as maintaining external fixation, switching to internal fixation using a plate (Figure 3), or ESIN [57,60,61,62]. When employed as the final option, external fixators have been linked with greater risks compared to alternative forms of fixation [57,61]. Open femoral fractures managed by external fixation required additional time to heal compared to alternative approaches, according to Hutchins et al. [57]. Fracture-related risks lengthened the duration required for fracture repair. Fifty percent of fractures in their cohort acquired osteomyelitis and twenty percent were malunited, making grade III fractures particularly challenging to treat. Intramedullary fixation is acquiring recognition in age-appropriate populations [30].

#### 3.2.3. Open Tibial Fractures

Open fractures of the tibia are the most prevalent injury in adolescents with multiple traumas and have received significant interest in the field of medicine [52,63,64]. A general consensus is that type II and type III fractures should be thoroughly debrided as swiftly as is feasible [65]. A variety of equipment, such as mono-lateral external fixators [66], circular frames [67], locking plates [64,68,69], ESIN [70], and combinations of fixators and implants, might be used to stabilize fractures. In type I and type II fractures, the recovery process becomes increasingly foreseeable, and closed reduction and immobilization appear to be equally effective [71,72]. Despite cast treatment being beneficial when dealing with scarce supplies, one ought to consider the advantages and disadvantages of keeping a child with multiple injuries in a limb cast, since additional wounds render mobilization problematic, thereby extending the hospitalization period. In accordance with soft tissue and loss of bone, fractures of type III perform in distinct ways, and the probability of developing an infection and nonunion is comparable to that of adults [30].

After tibial shaft fractures, compartment syndrome (CS) is a primary concern. Ho et al. [73] documented no instances of this condition among their patients who received a cast. Following closed reduction and casting, three patients (5.2%) experienced compartment syndrome according to Kinney et al. [74]. Both Kinney et al. [74] and Pennock et al. [75] determined that the prevalence of CS subsequent to the insertion of an elastic stable intramedullary nail (ESIN) was 2% and 4.5%, respectively. According to Pandya et al. [76], the incidence of compartment syndrome after ESIN appears to be as significant as 20%. Age, weight, mechanism of trauma, fracture pattern, and the existence of neurological disorders have been linked to a higher incidence of CS after ESIN [76]. Pennock et al. did not come across any instances of compartment syndrome resulting from open reduction internal fixation [75].

### 3.3. Hand Fractures

Pediatric hand fractures are the second most prevalent kind of fracture in children, having a yearly incidence ranging from 26.4 to 44.8 per 10,000 children [77,78]. Hand fractures often occur at two ages: toddlers and teenagers. Hand fractures in toddlers are typically the result of crush trauma or entrapment. The following spike in frequency arises around the ages of 13 and 15 and is associated with greater engagement in athletic endeavors [77,79].

Proximal/middle phalanx fractures constituted the most prevalent type (48%), according to one piece of research [80]; in a similar manner, Chew and Chong [79] identified a 49% incidence of proximal phalanx fractures in young people, while Lempesis et al. [78] identified a 60% frequency of phalangeal fractures in a comparable sample of individuals. Chew and Chong reported that the mean age of children who sustained fractures of the distal phalanges was 9 years, 12 years for the proximal phalanges, and 15 years for the metacarpals [79].

Pediatric hand fractures are seldom treated surgically [77]. Regardless of the fact that the frequency of hand fractures varies with age, the majority of extra-articular fractures are responsive to a closed reduction in every age category. Intra-articular fracture expansion has been linked with a lower likelihood of closed reduction therapy solely; therefore, open intervention is frequently necessary. Intra-articular fractures have a greater probability to require ORIF [81]. In such circumstances, approximately one-third of fractures among children over the age of five are going to need open therapy fixation, whilst over fifty percent of fractures in children under the age of five are going to need the same management.

### 3.4. Pelvic Trauma

Pelvic fractures are frequently caused by high-energy trauma and linked to limb, thorax, vertebrae, abdominal, and head trauma [56,82,83]. In contrast to adults, pelvic trauma is not a culprit of fatalities in children with multiple injuries. Nevertheless, related trauma to the cranium and organs can be deadly. Children with pelvic fractures are evaluated attentively and exhaustively to clear rectal and urogenital trauma, consisting of CT imaging in addition to suitable contrast investigations. A large percentage of pelvic fractures are steady injuries that can be effectively handled without surgery and promptly mobilized [84]. Pelvic binders, a C-clamp, or an external fixator might be used to stabilize problematic fractures. The latter is simple to handle and optimal if abdominal and urinary accessibility is required.

In pediatric trauma, the frequency of pelvic fractures varies between 0.8% and 1.6% [85,86], while mortality rates differ from 5% to 6.3%. Swaid et al. [85] evaluated a cohort of 99,579 children (0 to 14 years old) with blunt trauma and reported 812 (0.8%), with an average age of 8, with fractures in the pelvic region. The average age of children lacking pelvic fractures following blunt trauma was 6 years; consequently, this disparity in age warrants clinical evaluation and caution. Children with a fractured pelvis had an overall mortality rate of 5.2%, compared to 0.3% for those with no pelvic fracture. This fatality rate was generally linked to extensive traumatic head injuries. Kruppa et al. [87] compared unsteady, surgically managed pelvic ring injuries to solid, pelvic ring traumas, managed without surgery, in a retrospective study of 33 patients with an average age of 12.6 years. The research measured radiographic deformity, leg-length disparity, pain in the lower back, and sacroiliac joint discomfort as endpoints. Children with posterior sacral misalignment of 5 to 10 mm in either group experienced considerably greater distress than those with 0 to 4 mm displacement. Persistent deformity failed to improve. Thirteen children (39%) had persistent lower back/sacroiliac joint symptoms, with the incidence being substantially greater in the surgical group [87]. The results of this study aid in guiding conversations with families experiencing this trauma, but have no impact on medical practice. After analyzing 124 patients with pediatric pelvic fractures, Shore et al. [86] suggested an update to the Torode classification. Considering the greater force necessary to produce an anterior and posterior ring fracture in minors, the revised Torode PPF classification subdivided type III injuries into A (basic, stable anterior ring fractures) and B (“stable” anterior and posterior ring fractures) [86]. A total of 71% of those examined had type III injuries, and type III-B injuries were >2.5 times more probable to necessitate the transfusion of blood compared to the type III-A group. Across the patients with pubic rami injuries who underwent open reduction internal fixation (2 patients) or external fixation (EF) for stabilization (7 patients), 8 were skeletally developed [86].

### 3.5. Ankle Fractures

Fractures of the ankle in children are a widespread reason for orthopedic evaluation. Trauma to the ankle without radiographic confirmation of a fracture and soreness surrounding the physis of the distal fibula is frequently categorized as nondisplaced Salter–Harris type I physeal fractures of the distal fibula (SH1DF) [88]. Utilizing MRI, Boutis et al. [88] evaluated the incidence of SH1DF in radiographically negative ankle harm in a study including 135 children. Four children (3%) had SH1DF, while eighty percent had ligamentous lesions and twenty-two percent had bone injuries [88]. There was a concealed fibular avulsion fracture in 35% of the patients with ligamentous lesions. Employing an anterior talofibular ligament image or 4-week follow-up X-rays, Kwak et al. [89] determined that 26% of children with a confirmed ankle injury had a concomitant fibular avulsion fracture. The research conducted by Boutis et al. [88] also assessed the results one month following the treatment of every patient with a detachable ankle splint and their reinstatement to routines as permitted and discovered no statistically noteworthy distinction in scores across fractures and ligamentous trauma.

### 3.6. Foot Fractures

The vast majority of non-amputation traumas affect the lower limbs and feet [90]. Such wounds often necessitate comprehensive treatment, including frequent visits to the surgical department for irrigation, the removal of debris, and significant soft tissue covering.

Talus fractures are as uncommon in children as they are in adults, but they are significant due to the bone’s perilous blood supply and the probability of avascular necrosis that occurs in dislocated fractures. Talus fractures encompass neck, body, medial, or lateral process fractures, and osteochondral lesions. The breaking of the talus neck is the most frequent lesion in children [91].

Calcaneal fractures constitute almost 2% of adult fractures but are uncommon in young people. Os calcis fractures are responsible for one-third of all tarsal fractures in children and are associated with a more favorable outcome than in adults. Calcaneal fractures are most commonly caused by falls from higher levels. Injuries are caused by a mixture of axial forces and shear [91]. Overall, calcaneal fractures in children tend to be less serious, respond favorably to conservative therapy, and warrant surgery only infrequently [92,93]. Regardless of whether minor radiological anomalies, including a modest decrease in Bohler’s angle or degenerative alterations in the subtalar joint, are observed, subsequent functional outcomes are favorable [92].

Cuboid fractures are rare in children, but they merit being evaluated as the possible causes of a limping child with an ache in the outer portion of the foot and difficulty bearing pressure following a wound to the foot [91].

Tarsometatarsal injuries may occur as a consequence of either direct or indirect circumstances. Items landing on the foot generate direct harm, leading to a tear of the plantar ligaments. Metatarsals typically shift in a plantar orientation, resulting in significant soft tissue damage. Indirect wounds are considerably more prevalent and can occur as the outcome of a single or combined forceful plantar flexion or abduction action. These can be caused by a vertical force in plantar flexion, such as falling from an elevated position or attempting to brake pace with the foot when cycling. Comparable lesions can occur as an effect of heel-to-toe compression, as when the body’s weight falls onto the heel in the position of kneeling [91].

Items dropping on the foot or harming a toe are common causes of phalangeal fractures. Such wounds recover quickly, usually around 3 to 4 weeks. All that is necessary is to strap the wounded toe to its surrounding toe. Nevertheless, caution needs to be applied in order to prevent rotational misalignment. To prevent osteomyelitis, open phalange fractures demand extensive debridement and irrigation in combination with medication [91].

Seymour fractures are defined as distal phalangeal growth plate fractures with an overlaying nail bed injury [94]. The aforementioned injuries are classified as open fractures due to the rupture of the underlying nail bed. Seymour fractures can develop in either the toes or fingers and may result in serious complications if not identified and properly treated. In one retrospective assessment of five patients with open fractures of the great toe distal phalanx, no infection was observed to develop in those who were identified promptly and administered antibiotics. Nevertheless, in three instances with postponed assessment and therapy, the individuals suffered osteomyelitis [95]. Seymour fracture treatment includes removing the nail plate as well as any surrounding soft tissue to enable precise reduction. The area of fracture requires being carefully irrigated before repairing the sterilized matrix with 5-0 or 6-0 chromic gut stitches. The fracture is at this point in time steady and does not require fixing. If the fracture is still unsteady, retrograde K-wire fixation might be necessary [96].

### 3.7. Closed Multiple Fractures of Long Bones

Closed fractures of long bones in children with multiple traumas ought to be ideally surgically stabilized, despite the fact that they are typically managed non-operatively. With a lack of brain injury due to trauma, prompt stabilization lowers suffering, lessens the repercussions of immobilization [97], facilitates transport and mobilization, and shortens hospitalizations. Surgical fixation is recommended to be performed within the second and third days as long as the child is stable. Performing permanent fixation in the initial phases of multiorgan dysfunction is preferable. Long bone fractures warrant being managed vigorously, irrespective of extensive brain injuries, considering the children’s substantial capacity for recovery. A wide variety of devices and approaches are employed in order to stabilize fractures in children. Growth plates must be considered when making the fixation. For the humerus, forearm, femur, and tibia, intramedullary implants, primarily malleable nails, are acquiring recognition. They are advantageous due to their low-risk method, limited bleeding, and simple and swift placement. Disadvantages consist of metaphyseal fractures, certain of which are communicable, segmental shaft fractures, and taking into account the person’s weight. Negative aspects encompass discomfort coming from the nail tips at the point of entrance and the requirement for withdrawal and re-fracture in the event the nail is pulled out too soon. When avoiding piriform fossa entry, reamed intramedullary nails are preferable for adolescents and provide higher support [30]. Plating is a great alternative for fractures that cannot be treated with malleable nails and for metaphyseal fractures. External fixators tend not to be optimal for the permanent treatment of fractures, considering recovery from fractures may become slow-moving and pin-tract infection escalates throughout the course of time. Fixators are additionally consistently correlated with misalignment compared to various other techniques. Intraarticular, physeal, and distal radial fractures may be treated with K-wires or cannulated screws [30].

### 3.8. Uncommon Fracture Types

Monteggia and Galeazzi fracture-dislocations can go undetected or overlooked due to inappropriate X-ray images or poor analysis, resulting in suboptimal functionality when they are not correctly identified and managed [98]. Four highly uncommon forms of Monteggia fracture-dislocation have been defined as Monteggia subtypes [99]. Every single instance of Mikic’s documented Galeazzi fracture dislocations was handled conservatively [100]. Fowles and Kasab outlined neck-of-radius fractures as being rare and proposed prompt identification and precise reduction and fixation [101]. Gaston demonstrated that suitable closed techniques yield superior outcomes to open procedures for this kind of injury [102]. In order to repair unsteady epiphyseal lesions of the radial head, Metaizean proposed an oblique K-wire [103]. A flexion-type supracondylar humerus fracture is likewise uncommon, representing 2% to 3% of all reported supracondylar fractures. Wilkins suggested open reduction and internal fixation in order to treat this kind of fracture [104]. Regarding supracondylar humerus fractures with ipsilateral forearm bone fractures, C L Stanitski characterized them as relatively rare and stated that every one of these ipsilateral injuries warrants individual treatment [105]. Vialle R. et al. showed that anterior hip dislocation is significantly far less prevalent compared to posterior hip dislocation, with the majority of them being treated with a closed reduction [29]. A fracture of the femoral neck in a child is a somewhat unusual injury, contributing to only 1% of all pediatric fractures, and they generally result from high-energy trauma [106]. In adolescents, patella fractures are exceedingly infrequent [107]. In children, tears of the patellar tendon are atypical but possible [108]. Fracture detachment of the upper tibial epiphysis seldom occurs. Waston-Jones categorized this injury into three distinct groups [109]. Fracture of the talus appears to be brought on by forced dorsiflexion, which arises far less frequently in minors than in adults. Closed reduction and casting have been proposed as adequate management by Boyed and Knight [29]. Pelvic fractures with Morel-Lavallée soft tissue lesions (MLL) are an atypical occurrence; therefore, treatment remains under debate [110].

## 4. The Medico-Legal Approach to Traffic Accidents in Children and Adolescents

The rate of fatalities from RTAs is an important matter of public health that has considerable social and financial implications. In urban areas, children are particularly susceptible to pedestrian-related casualties. The evaluation of different risk variables for mortality among young people could be helpful in the formulation of risk categories in RTAs as well as offering data regarding passenger safety strategies aimed at children and their parents [12]. Research studies examine diagnostic prospects and explore how to provide the most efficient medical care; nevertheless, forensic pathologists confront the most grievous impacts in deadly instances when mortality occurs often at the site of injury with little prospect of surviving. Assessment of the nature of injuries in fatal incidents on a global scale would enable an improved comprehension of the pathophysiology of accidental injuries and could additionally help develop precautionary measures [11]. The medico-legal approach to traffic accidents in children and adolescents is an important field that involves the intersection of medicine and law. When children and adolescents are involved in traffic accidents, there are specific considerations that need to be taken into account to ensure their well-being and address any legal implications. When focusing specifically on children and adolescents, it is crucial to understand the risk factors associated with severe injuries in road traffic accidents. A study conducted in Singapore aimed to describe the characteristics of injuries, the usage of restraints among road users, and the risk factors associated with severe injuries for children involved in traffic accidents. The study included children below 16 years old who presented to the emergency departments within 24 h after the injury. The study found that most road injuries involved motor vehicle occupants, but child pedestrians, bicyclists, and motorcyclists were more likely to sustain severe injuries compared to motor vehicle passengers. The study also highlighted that a significant proportion of bicyclists and motor vehicle passengers were not using helmets or restraints, respectively. The findings of this study emphasize the vulnerability of child pedestrians, bicyclists, and motorcyclists to severe injuries in traffic accidents. To address these issues, the study suggests that injury prevention efforts should focus on the enforcement of legislation to protect these high-risk groups. This could include measures such as promoting the use of helmets for bicyclists and improving compliance with seat belts and child restraint laws for motor vehicle passengers [111].

Pattern injuries to pedestrians in traffic accidents also play a significant role in medico-legal assessments. These injuries can be categorized into primary impact injuries, secondary impact injuries, secondary or tertiary injuries, and run-over injuries. Primary impact injuries occur as a result of the first impact between the victim and the vehicle. Secondary impact injuries involve subsequent impacts by the same vehicle, while secondary or tertiary injuries occur when the victim is struck by another vehicle or object. Run-over injuries, on the other hand, happen when a vehicle runs over a part of the victim’s body. The specific body parts affected depend on the position of the pedestrian and the vehicle during the accident. Understanding these patterns helps in determining the severity and location of injuries sustained by children and adolescents involved in traffic accidents [112]. Factors such as the position of the pedestrian, the point of impact on the vehicle, and the braking behavior of the vehicle also influence the injuries sustained by pedestrians. For example, a pedestrian’s body part that suffers an injury depends on whether they were standing, walking, or lying on the road during the accident. The point of impact on the vehicle, whether it is the front or side, also affects the injuries. In cases where the vehicle brakes violently during the impact, the front end of the vehicle dips down, potentially leading to lower limb injuries in pedestrians [112]. The medico-legal aspects of RTAs involving children and adolescents present unique challenges. The unfortunate loss of a child’s life in a traffic accident can lead to judicial consequences for the adult responsible for driving the vehicle, carrying the child, or ensuring the proper use of safety devices. In cases where a child’s death occurs due to a traffic accident, the responsible adult may face legal proceedings that compound the family’s tragedy. Analyzing court cases and integrating competencies from various fields can provide insights into the medico-legal aspects of these accidents [113]. One crucial aspect of the medico-legal approach to traffic accidents in children and adolescents involves the responsibility of drivers and caregivers for the proper use of safety measures. Child safety seats and restraint systems play a vital role in protecting children in vehicles. The responsibility of drivers to ensure the appropriate use of child safety seats and the efficacy of these restraint systems are subjects of discussion in the medico-legal context. Analyzing specific cases and evaluating the effectiveness and limitations of child restraint systems can contribute to addressing these concerns [113].

Additionally, it is important to consider the psychological effects of RTAs on children and adolescents. These accidents can have significant psychological impacts on young individuals, leading to mental disorders and emotional distress. While there have been efforts to assess and address physical injuries resulting from traffic accidents, mental disorders, and their medico-legal assessment have received less attention. Understanding the relationship between RTAs and mental health outcomes is crucial for providing comprehensive support to affected children and adolescents [114]. Research on risk factors associated with RTAs involving children and adolescents is another important area of investigation. Factors such as gender, age, and socioeconomic status have been identified as potential risk factors. For example, male gender, younger age, and low income have been associated with an increased risk of road traffic accidents. Children from families in a lower socioeconomic group are roughly twice as probable as those from high-income families to be part of an accident [115]. Several hypotheses have been suggested: larger distances are driven (due to residing in the “periphery” distant from urban centers of employment) [116], driving less road-worthy vehicles, and associated risk practices, including neglecting to utilize seatbelts on their children, using mobile phones whilst driving, and disregarding traffic signals on the road [117]. Such research findings can help inform preventive measures and interventions to reduce the occurrence of accidents and mitigate their impact on young individuals [118]. From a legal perspective, the relationship between age and the violation of traffic laws is a critical consideration in the medico-legal approach to traffic accidents involving children and adolescents. Efforts are being made in various countries, such as Japan, to develop road traffic environments that prioritize the safety and security of children. These initiatives include implementing speed limits and physical measures to ensure safer pedestrian spaces and residential streets [119].

The American Academy of Pediatrics (AAP) has developed guidelines and policies on the most secure methods of transportation for children of all ages [120]. In order to minimize trauma in crashes involving motor vehicles, the AAP proposes car-restraint mechanisms tailored to age, weight, and/or height that lower expulsion from the vehicle, spread energy from the impact to bones instead of soft tissues, and decrease engagement of the head and appendages with the internal structure of the car [120,121]. When children outgrow their front-facing child safety position, they ought to switch to a belt-positioning booster position, according to recommended standards. They should continue to sit in the booster seat up until their vehicle’s lap and shoulder straps suit correctly, which is usually around the ages of 8 and 12 or until they achieve a height of 4 feet 9 inches [120,122]. Most parents follow standard car seat safety regulations for newborns and young children. Nevertheless, when children migrate to forward-facing child safety seats and booster seats, fewer are correctly secured, and more are inappropriately switched to an adult safety belt, leaving them exposed to significant harm in the event of an accident [121,123,124]. In the unforeseen case of a collision, the adults responsible for the children’s protection bear responsibility for positioning the child in an unsuitable spot inside the car or inadequately fastening them using an unsuitable seat belt. Usually, this obligation rests on the child’s parents or guardians. Disregarding these critical safety precautions might result in legal and liability consequences for the adults concerned, especially if their conduct or errors lead to the child’s injury during the collision [125,126].

## 5. Future Directions Regarding Child Pedestrian Motor Vehicle Collision Prevention

The US Federal Highway Administration identifies collisions, traffic density, and road conditions as crucial traffic safety information [127]. Every one of them is an impediment to overcome regarding PMVC [128]. Due to a dearth of precise information, the overall number of PMVC-related fatalities and injuries can easily be understated. For instance, it may prove difficult to estimate the unit of measurement for percentage estimation due to constraints in the source of information, including incorrect classification or discrepancies in accident timing and place [129]. The primary information providers for PMVC, especially those concerning minors, are reports from law enforcement and hospital documentation. Nevertheless, there is substantial evidence of inadequate reporting in police files, especially regarding accidents comprising fewer grievous casualties [130,131]. Furthermore, in a number of countries, law enforcement reports and medical records fail to include the precise spot of the accident, which is essential for preventive measures [132,133], with the insufficient disclosure of collisions is a significant problem, especially in low-/middle-income countries [134,135].

Future directions involve integrating pedestrian detection and collision avoidance systems in vehicles, improving vehicle crashworthiness, and promoting the use of child restraint systems. Emerging technologies have the potential to revolutionize child PMVC prevention by exploring the use of artificial intelligence, computer vision, and sensor-based systems to detect and alert both drivers and pedestrians about potential collision risks [136,137]. Enhancing the safety of pedestrian environments is crucial in reducing child PMVC. Future efforts should focus on implementing traffic calming measures, improving road infrastructure, enhancing visibility through better street lighting, and creating pedestrian-friendly zones [138]. Future strategies should include promoting driver education programs, enforcing traffic laws, raising awareness about the presence of child pedestrians, and implementing advanced driver assistance systems (ADAS) that prioritize pedestrian safety. To facilitate progress in child PMVC prevention, a collaboration between researchers, policymakers, urban planners, healthcare professionals, and community stakeholders is essential [139]. Future efforts should focus on establishing robust data collection and sharing systems, conducting multi-disciplinary research, and fostering partnerships to implement evidence-based interventions. The design and safety features of vehicles can play a significant role in reducing child PMVC.

Child PMVC disproportionately affect socio-economically disadvantaged communities. Communities with lower socioeconomic levels have a greater incidence of child pedestrian–vehicle collisions [140,141,142,143,144,145]. Additional factors indicating a low socioeconomic background, such as single-parent households and greater residence unsteadiness, have been found to be linked with a greater probability of child PMVC [146,147]. Among minors, socioeconomic discrepancies in pedestrian collision rates have proven higher than in adults [148]. In Toronto, Canada, minors residing in neighborhoods with the lowest median earnings experienced over five-fold the amount of PMVC leading to trauma in comparison with minors inhabiting areas with the greatest revenue [145]. A separate analysis conducted in Ontario, Canada, concluded that whereas hospitalizations for child pedestrian collisions dropped by 18% province-wide between 2008 and 2015, the majority of this decline was recorded in communities with greater financial resources, with a minor, insignificant improvement recorded in areas with lowest earnings [149]. Reports of escalated child pedestrian harm rates appear to be continuously related to assessments of reduced income, employing a variety of analysis strategies, economic standing regulations involving individualized and area-based measurements, and contexts. Nevertheless, the underlying cause of increased risk continues to be insufficiently comprehended. Future directions should prioritize addressing these disparities by implementing targeted interventions, enhancing accessibility to safe pedestrian infrastructure, and providing educational programs on road safety for children and families in vulnerable populations [150].

Addressing traffic-related hazards, multiple studies have demonstrated that the efficacy of both educational and precautionary traffic safety initiatives remains disputed [151,152]. Advertising and educational initiatives promoting road safety focus mainly on encouraging drivers to adhere to legal speed limitations (by presenting them with knowledge regarding the repercussions of speeding, for instance). While there is currently no general agreement on the efficacy of road safety prevention measures, data indicate that advertisements that emphasize warnings (pointing out hazards, including getting wounded or killed in a collision) are highly successful [153].

The Sustainable Development Goals of the United Nations [154] encompass a strategy regarding neonatal, child, and young adult health. The proposed approach shows that the healthcare priority in the Central American region faces considerable obstacles with regard to the legislation and funds to enhance and promote a more secure urban environment, less hazardous drivers, and more reliable transportation. The Central American Traffic Safety Manual is the result of a coordinated attempt by the six nations with Spanish-speaking populations to formulate uniform, unambiguous, and customized road safety standards [155]. This guide provides a prospect for Central American nations to establish, enforce, and harmonize legislation regarding road safety. The introduction of child retention systems (CRSs) is a particular preventive strategy for MVC-related trauma in children. Research has shown that proper implementation of CRSs decreases fatalities among infants and children, as well as trauma magnitude and frequency, and therefore accident-related consequences and impairment in this highly susceptible group [156,157,158,159,160]. Although Costa Rica has enacted a regulation requiring the application of CRSs, Panama and Guatemala do not possess laws for CRSs that conform with worldwide requirements [26].

It is important to note that the above future directions are based on the existing literature and may evolve as new research and technologies emerge. Continued research, policy development, and community engagement are crucial to ensure the implementation of effective strategies that reduce child pedestrian motor vehicle collisions and safeguard the well-being of children.

## 6. Conclusions

Traffic accidents in children and adolescents present a complex and multifaceted challenge that requires a comprehensive medico-legal and orthopedic approach. Understanding the epidemiology, risk factors, injury patterns, and consequences associated with such accidents is crucial for developing effective prevention strategies, implementing appropriate legal measures, and providing optimal medical care. By addressing these aspects, society can work towards reducing the incidence and severity of traffic accidents in children and adolescents, ultimately ensuring their safety and well-being on the roads. The orthopedic approach in traffic accidents involving children and adolescents encompasses comprehensive assessment, appropriate treatment strategies, and focused rehabilitation efforts. By addressing the specific needs of young patients, orthopedic specialists play a vital role in optimizing outcomes and facilitating recovery from the orthopedic injuries caused by traffic accidents. The medico-legal approach to traffic accidents in children and adolescents involves understanding the outcomes of such accidents, conducting personal injury assessments, identifying risk factors associated with severe injuries, and analyzing pattern injuries to pedestrians. Guidelines for personal injury assessment provide a comprehensive framework for evaluating trauma outcomes. Moreover, efforts should focus on enforcing legislation, promoting the use of restraints and protective gear, and targeting injury prevention strategies for vulnerable groups, such as child pedestrians, bicyclists, and motorcyclists. By considering these factors, the medico-legal approach can contribute to improving the understanding, prevention, and management of traffic accidents in children and adolescents.

Addressing child pedestrian motor vehicle collisions requires a multidimensional approach involving prevention strategies, technological advancements, infrastructure improvements, and educational interventions. By focusing on these future directions, we can work towards creating safer environments for child pedestrians and reducing the risk of PMVCs.

## Figures and Tables

**Table 1:** Open fracture complications in children who suffered road traffic accidents.

Local Complication			Systemic Complications
Urgent	Less urgent	Late	
Local visceral injury	Fracture blister	Delayed union	Hypovolemic shock
Vascular injury	Plaster sores	Malunion	Fat embolism syndrome
Nerve injury	Pressure sores	Nonunion	ARDS
Compartment syndrome	Nerve entrapment	Avascular necrosis	Infection
Infection	Myositis ossificans		
Gas gangrene			

## Data Availability

Not applicable.
